# Predicting students’ academic progress and related attributes in first-year medical students: an analysis with artificial neural networks and Naïve Bayes

**DOI:** 10.1186/s12909-023-04918-6

**Published:** 2024-01-19

**Authors:** Diego Monteverde-Suárez, Patricia González-Flores, Roberto Santos-Solórzano, Manuel García-Minjares, Irma Zavala-Sierra, Verónica Luna de la Luz, Melchor Sánchez-Mendiola

**Affiliations:** 1 Coordination of Open University, Educational Innovation and Distance Education, (CUAIEED), National Autonomous University of Mexico (UNAM), Mexico City, Mexico; 2 Faculty of Medicine, National Autonomous University of Mexico (UNAM), Mexico City, Mexico

**Keywords:** Educational data mining, Student academic progress, Prediction, Medical education, Artificial neural networks, Naïve Bayes

## Abstract

**Background:**

Dropout and poor academic performance are persistent problems in medical schools in emerging economies. Identifying at-risk students early and knowing the factors that contribute to their success would be useful for designing educational interventions. Educational Data Mining (EDM) methods can identify students at risk of poor academic progress and dropping out. The main goal of this study was to use machine learning models, Artificial Neural Networks (ANN) and Naïve Bayes (NB), to identify first year medical students that succeed academically, using sociodemographic data and academic history.

**Methods:**

Data from seven cohorts (2011 to 2017) of admitted medical students to the National Autonomous University of Mexico (UNAM) Faculty of Medicine in Mexico City were analysed. Data from 7,976 students (2011 to 2017 cohorts) of the program were included. Information from admission diagnostic exam results, academic history, sociodemographic characteristics and family environment was used. The main dataset included 48 variables. The study followed the general knowledge discovery process: pre-processing, data analysis, and validation. Artificial Neural Networks (ANN) and Naïve Bayes (NB) models were used for data mining analysis.

**Results:**

ANNs models had slightly better performance in accuracy, sensitivity, and specificity. Both models had better sensitivity when classifying regular students and better specificity when classifying irregular students. Of the 25 variables with highest predictive value in the Naïve Bayes model, percentage of correct answers in the diagnostic exam was the best variable.

**Conclusions:**

Both ANN and Naïve Bayes methods can be useful for predicting medical students’ academic achievement in an undergraduate program, based on information of their prior knowledge and socio-demographic factors. Although ANN offered slightly superior results, Naïve Bayes made it possible to obtain an in-depth analysis of how the different variables influenced the model. The use of educational data mining techniques and machine learning classification techniques have potential in medical education.

## Background

Dropout and poor academic performance are two persistent problems in medical schools in emerging economies. Recent studies have found that these issues have a higher prevalence during the first academic year and have an important correlation with a student’s likelihood to graduate [1–3]. An analysis of medical students’ trajectories with data from 25 cohorts (1985 to 2010) at the National Autonomous University of Mexico (UNAM) Faculty of Medicine confirmed similar trends: 54% of the students successfully completed all the first year required courses, and only 41% graduated at the end of the established six-year period in the curriculum [4].

Universities have a deep interest in analysing student data to learn more about how they can increase retention, decrease dropout rates and enhance students’ academic performance. Pertinent data include academic background, sociodemographic and economic profiles, students’ academic history, grades, among others [5, 6]. Research carried out in Mexican universities and around the world have found that academic progress of medical students is strongly correlated with their prior academic success [6–13]. Other important factors include socio-economic status, English proficiency and age; this last factor has been found to be indirectly correlated with students’ academic success [14–17].

Identifying as early as possible students that are at-risk for delay or interruption of their academic trajectory, and knowing the factors that contribute to their success would be useful for designing interventions to promote academic achievement throughout the program. Implementing data-informed actions could increase the number of students that successfully finish the first academic year, and reduce the social, institutional, emotional and personal costs that are associated with dropout [18]. The possibilities and advantages of identifying in a timely manner students at risk of dropout have motivated educational institutions and researchers to assess different tools and methodologies that could make this possible [19, 20].

### Educational data mining (EDM)

Educational Data Mining (EDM) methods can help in the early detection of students at risk of poor academic progress and dropping out, can enhance the personalization of learning, and are key for visualizing academic data in a variety of ways. Recently, Pawar & Jain [21] reported in a systematic review that techniques of data mining and artificial intelligence (IA) are effective to predict student performance.

EDM research studies have explored the performance of different statistical and machine learning models for prediction tasks, such as artificial neural networks, decision trees, logistic regression, Naïve Bayes, Bayesian networks, vector support machines, extreme learning machines, k-nearest neighbor, k-means clumping algorithm, J48, zeroR, random trees [22–31]. The most accurate predictions have been achieved with vector support machine models, k-nearest neighbor, artificial neural networks, Naïve Bayes and random forest.

Despite the advances in this area, studies conducted with EDM in medical education are scarce. In other countries, research has focused on using data collected from Learning Management Systems (LMS) or digital resources, to identify patterns of online behaviour and predict performance [32, 33]. In computer science and engineering, several studies about prediction of students’ performance with EDM have been carried out [34, 35].

The main goal of this study was to measure the performance of two machine learning models, Artificial Neural Networks (ANN) and Naïve Bayes (NB), to classify first year medical students according to their attributes into regular (when the student completes the credits required by the program in each cycle and is promoted to the next curricular year) or irregular students, using sociodemographic data and academic history. An additional goal was to identify the most important factors in the models.

## Methods

This study followed the general knowledge discovery process: pre-processing, data analysis, and validation. The following sections detail the process for each phase.

### Preprocessing

#### *Data sources*

The undergraduate medical program at the National Autonomous University of Mexico Faculty of Medicine has a large student population, 10,104 students in 2022 [36]. When students apply for admission, information related to their family environment, socio-economic status and prior academic trajectory is collected through a questionnaire. After students are enrolled in the program, their knowledge in eight subjects is assessed using a standardized multiple-choice question (MCQ) diagnostic exam. During the program, data on their progress is recorded (grades, type of final exams -regular or resit^1^- and credits achieved). At the end of each academic year, students require a specific number of credits in order to be able to enrol in next year’s courses. Students that have less than the required credits have to enrol again in the courses they failed, and they cannot be promoted to the next curricular year, so they lose a year and are delayed in their academic trajectory.

Students were categorised as either regular or irregular, based on the number of credits they obtained during their first academic year:

- Regular: students who successfully completed all the required courses for the first year and obtained all the required credits at that time (value 1).
- Irregular: students who fail one or more of the required courses for the first year, and consequently do not have all the required credits (value 0).

#### *Data characteristics*

This study used data from 7,976 anonymized students from the 2011 to 2017 cohorts of the program. It included information from the students’ diagnostic exam results, academic history, sociodemographic characteristics and family environment [12, 37, 38]. The main dataset included 48 variables (24 categorical, 8 discrete numerical, and 16 continuous numerical). Table 1 contains all the variables in the dataset classified in different groups: student’s demographics, family environment, socio-economic status, prior educational experience, type of admission and student progress.

**Table 1:** Explanatory variables collected at admission to the medical school (UNAM Faculty of Medicine, Mexico City)

Group	Explanatory variables
**Students’ demographics**	Gender (SEX), age at admission (INITIAL_AGE), marital status (MARITAL_STATUS), children (CHILDREN), employment (STUDENT_EMPLOYMENT).
**Family environment**	Whether the student is the first, second or third born (NUM_SIBLINGS), academic background of the father (FATHER_STUDIES), academic background of the mother (MOTHER_STUDIES), father’s occupation (FATHER_OCCUP), mother’s occupation (MOTHER_OCCUP).
**Socio-economic status**	Students’ home (HOME), number of rooms at home (ROOM_NUM), number of lightbulbs in home (NUM_FOCOS), number of people that live in home (LIVE), number of family members that work (NUM_WORK), persons that financially support the student (FIN_SUPPORT), number of financially dependent persons in the students’ family (DEPENDENT), monthly family income (MONTH_INCOME).
**Prior educational experience**	Type of primary school, public, private or both (PRIMARY), type of junior high school public, private or both (JUNIOR_HIGH_SCHOOL), type of high school public, private or both (HIGH_SCHOOL), high school subsystem (SUBSYSTEM), high school shift (SHIFT), timely completion of high school (three years) (HIGHSCHOOL_THREEYEARS), remedial exams in high school (EX_EXAMS), high school grade average (AVERAGE_HIGHSCHOOL), student’s perception of academic success (SUCCESS), institutional affiliation of high school (AFFILIATION), high school campus (HS_CAMPUS).
**Type of admission**	Type of admission (TYPE_ADMISSION).
**Student progress**	Percentage of obtained credits at the end of the first year (PROGRESS). Students were regular or irregular at the end of the first year if they obtained 100% of credits or not (ACADEMIC_STATUS_1STY).

Table 2 lists the groups of variables from the students’ performance in the admission diagnostic exam: students' scores in eight high school subjects, including proficiency in Spanish and English. From this point forward, we will use the term students’ prior knowledge to refer to this group of variables.

**Table 2:** Variables from scores in the admission diagnostic exam (UNAM Faculty of Medicine, Mexico City)

Group	Explanatory variables
**Prior academic achievement**	Percentage of correct answers in admission diagnostic test: global score (PGLOBAL), mathematics (PMAT), chemistry (PCHE), physics (PPHYS), biology (PBIO), geography (PGEO), Mexican and world history (PMXHIS and PWHIS), literature (PLIT).
**Spanish proficiency**	Percentage of correct answers in diagnostic test of Spanish proficiency at admission: global (PESP), reading comprehension (PRC), vocabulary (PVOC), spelling (PSPE), grammar (PGR).
**English proficiency**	Student’s English proficiency according to the Common European Framework: does not qualify, basic level A1, basic level A2 and intermediate level B1 (ENG_LEVEL); percentage of correct answers in diagnostic test of English proficiency (PENG).

The dependent variable (ACADEMIC_STATUS_1STY) was calculated using the percentage of credits completed at the end of the first year (PROGRESS).

#### *Dataset preparation*

From the initial 7,976 records, 910 (11.4%) were excluded from analysis because they had a significant percentage of missing data (they had little or no information in their demographics survey or they did not take the diagnostic exam). There was a slight difference in the class distribution, 47.8% of the students were categorised as irregular and 52.7% as regular. The EDM models used in this study had different data pre-processing requirements. For the Naïve Bayes model, numeric variables were converted to categorical in order to have a more balanced distribution of the number of students that belong to an attribute possible values. For example, a variable that reflected a grade would have few students that had a specific numeric value, compared to the number of students that would be within a range of the grade (e.g., 50–60%). This conversion helps in the interpretation of how different values influence the model as well as improve the model’s accuracy [39, 40].

For both models, the initial dataset was divided into a “training set” consisting of 80% randomly selected student records, and a “test set” with the remaining 20%. This distribution was chosen arbitrarily trying to balance the models’ accuracy and to avoid overfitting.

#### *Artificial neural networks*

The categorical variables were converted to numerical values by applying one-hot encoding, which separates the categories within each variable and transforms them to dichotomous variables with a value of 1 if the attribute is available and 0 if it is not [41]. Missing values were replaced using a smooth imputation with the SimpleImputer library of Scikit-learn in Python. In the case of numerical variables, missing values were replaced by the mean; in categorical variables, the mode was used since the percentage of missing values was less than 10% [42].

#### *Naïve Bayes*

Continuous numeric variables associated with percentages were categorised in five groups using percentile values as a reference [43]. Categories for discrete numeric variables were redefined so that each one contained an even number of cases. Since missing values ​​were treated as a possible value for the variables, imputation techniques were not used.

### Data analysis

#### *Data mining models*

The ANN and the Naïve Bayes models were selected due to their reported high performance in classification tasks [39, 44]. Two classification tasks were carried out in both models: one to predict students’ regularity and the other to predict their irregularity. Even though trying to predict both scenarios might be redundant, this was done to explore if there would be any difference in the models regarding the results and the influence of the predicting variables.

#### *Artificial neural networks*

ANNs are a machine learning algorithm inspired by the physiology of neurons [27], in particular, how a neuron transmits an impulse based on its different connections. In the model, a neuron is a unit that will output a numeric result by computing the different weights, input values and an arbitrary bias through an activation function [27]. For this study, a Multilayer Perceptron (MLP) neural network with backpropagation (BP) with two hidden layers was used. The models were created using Python Scikit-learn library for data management and Google’s TensorFlow using the Keras interface library for setting up and running the models. The ANNs were fine-tuned based on their accuracy, specificity and sensitivity.

A disadvantage of this model is that ANNs are considered “black boxes”, it is impossible to dissect and understand exactly how the network produces a determined result or how each variable influences it [27]. However, there are some methodologies that can estimate the influence of each variable on the model such as the sensitivity analysis. A series of datasets were prepared where a variable was removed from each dataset, then multiple ANN were trained and their accuracy obtained through cross validation. Afterwards, the variables were ranked based on how much subtracting them from the dataset influenced the accuracy of the model.

#### *Naïve Bayes*

NB is a probabilistic classification method adequate for data sets with a high number of variables. As its name implies, it is based on Bayes’ theorem and assumes that the predictive variables are not conditionally dependent. It estimates the post-probability of an event or condition given the values of the predictive variables [45].

The model was created using the R programming language. First, the probability of belonging to a class (e.g. regularity) for each variable possible value was calculated. Second, a score was estimated for each variables’ values considering their likelihood of belonging to the target class. Third, the score for each student was obtained by adding the individual score of each variables’ value based on their data. Finally, an analysis was carried out to select the best score threshold for classifying a student. A ROC curve analysis was carried out to determine the optimal score threshold. Multiple models were trained with different thresholds (from − 9.73 to 8.48) to determine if a student was at-risk or not. The best value for the threshold (0.43) was determined by considering the models’ sensitivity and false positive rate.

In contrast with ANNs, with the Naïve Bayes model it is possible to analyse the influence that each variable has in the model based on its predictive value [42]. In order to better understand the significance of each variable and its values, the *epsilon* values were calculated:

$${\epsilon}_{{X}_{i}}=\frac{{N}_{{X}_{i}}[P\left({X}_{i}\right)-P\left({C}_{k}\right)]}{{\left[{N}_{{X}_{i}}P\right({C}_{k}\left)\right(1-P\left({C}_{k}\right)]}^{1/2}}$$

Where ${C}_{k}$ represents the class, ${X}_{i}$ the attribute in accordance with the response category and ${N}_{{X}_{i}}$the number of students with attribute ${X}_{i}$. Categories with *epsilon* values greater than 2 or lower than − 2 are considered significant for prediction [42, 43].

### Validation

Both models were validated using a test dataset to assess how they would perform with new data. The models were analysed based on their accuracy, sensitivity, specificity, positive predictive value, and negative predictive value. These parameters were used because of their usefulness for designing interventions. They provide a better understanding of the models’ limitations and how they can be implemented in an individual or population scale.

Accuracy represents the percentage of correct classifications achieved by a model.

$$accuracy=\frac{Correctly\;classified}{Total\;population}$$

Specificity indicates the percentage of students *that do not belong* to the target class and were classified *correctly* by the model.

$$specificity=\frac{True\;negatives}{Total\;negatives}$$

Sensitivity denotes the percentage of students *that belong* to the target group and were classified *correctly* by the model.

$$sensitivity=\frac{Positives\;correctly\;classified}{Totalpositives}$$

The positive predictive value indicates the probability that a student *belongs* to the target group given that the model predicted they belong to it.

$$ppv=\frac{True\;positives}{Total\;positives}$$

The negative predictive value is the probability that a student does *not belong* to the target group given that the model did not classify them as such.

$$npv=\frac{True\;negatives}{Total\;negatives}$$

## Results

### Models’ performance

The ANNs models had a slightly better performance in terms of accuracy, sensitivity, and specificity. A few differences were observed when trying to predict regular students vs. irregular students between the models. The ANN achieved a slightly better accuracy while predicting irregular students (Table 3). This could partially be explained by the imbalance of both classes in the dataset (47.8% irregular students vs. 52.7% regular students).

**Table 3:** Results obtained from the models for both classes (regular and irregular students). *N* = 7,066 from 2011 to 2017 cohorts, UNAM Faculty of Medicine, Mexico

Target groups	Model	Accuracy	Sensitivity	Specificity	PPV	NPV
**Irregularity**	Neural networks (ANN)	0.74	0.72	0.75	0.79	0.68
	Naïve Bayes	0.71	0.72	0.70	0.70	0.72
**Regularity**	Neural networks (ANN)	0.73	0.75	0.72	0.67	0.79
	Naïve Bayes	0.71	0.70	0.72	0.72	0.70

*PPV* positive predictive value, *NPV* negative predictive value

### Factors that influence students’ academic progress

In the case of the ANNs, sensitivity analysis showed that none of the variables had a difference in accuracy greater than 0.02 when removed from the dataset. Most of the models achieved accuracy values between 0.72 and 0.74, the slight variations could imply that the model depends on a combination of variables rather than a single variable.

A review of the 25 variables with highest predictive value in the Naïve Bayes model showed that the percentage of correct answers in the diagnostic exam (PGLOBAL) was the best variable for predicting regular or irregular students. Table 4 lists the top 10 variables based on their predictive values. Other relevant variables were in the following groups: (i) prior academic achievement (high school grade average, percentage of correct answers in the diagnostic exam in mathematics, chemistry, biology, English proficiency, physics, Spanish proficiency, grammar, literature, world history, geography and spelling,); (ii) school where they previously studied (high school subsystem, high school campus, high school shift, type of primary school, type of junior high school public) and (iii) the parents’ academic background.

**Table 4:** Best predictive variables based on the Naïve Bayes model analysis

#	Regularity	Irregularity
1	PGLOBAL - Percentage of correct answers in diagnostic test at admission: global score - greater than or equal to 65%	PGLOBAL - Percentage of correct answers in diagnostic test at admission: global score - less than 43.3%
2	PMAT - Percentage of correct answers in diagnostic test at admission: mathematics - greater than or equal to 59.4%	SUBSYSTEM - High school subsystem - B
3	PCHE - Percentage of correct answers in diagnostic test at admission: chemistry - greater than or equal to 71.4%	PCHE - Percentage of correct answers in diagnostic test at admission: chemistry - less than 43.7%
4	PBIO - Percentage of correct answers in diagnostic test at admission: biology - greater than or equal to 75%	PBIO - Percentage of correct answers in diagnostic test at admission: biology - less than 46.7%
5	PENG - Percentage of correct answers in diagnostic test of English proficiency - greater than or equal to 90%	PENG - Percentage of correct answers in diagnostic test of English proficiency - less than 50%
6	ENG_LEVEL - Student’s English proficiency according to the Common European Framework -B1	ENG_LEVEL - Student’s English proficiency according to the Common European Framework - Does not classify
7	PPHYS - Percentage of correct answers in diagnostic test at admission: physics - greater than or equal to 64.3%	PMAT - Percentage of correct answers in diagnostic test at admission: mathematics - less than 32.3%
8	PESP - Percentage of correct answers in diagnostic test of Spanish proficiency at admission: global - greater than or equal to 74.6%	PPHYS - Percentage of correct answers in diagnostic test at admission: physics - less than 37.5%
9	PGR - Percentage of correct answers in diagnostic test of Spanish proficiency at admission: grammar - greater than or equal to 75%	PESP - Percentage of correct answers in diagnostic test of Spanish proficiency at admission: global - less than 53.3%
10	PLIT - Percentage of correct answers in diagnostic test at admission: literature - greater than or equal to 80%	HS_CAMPUS - High school campus

**Fig. 1:**
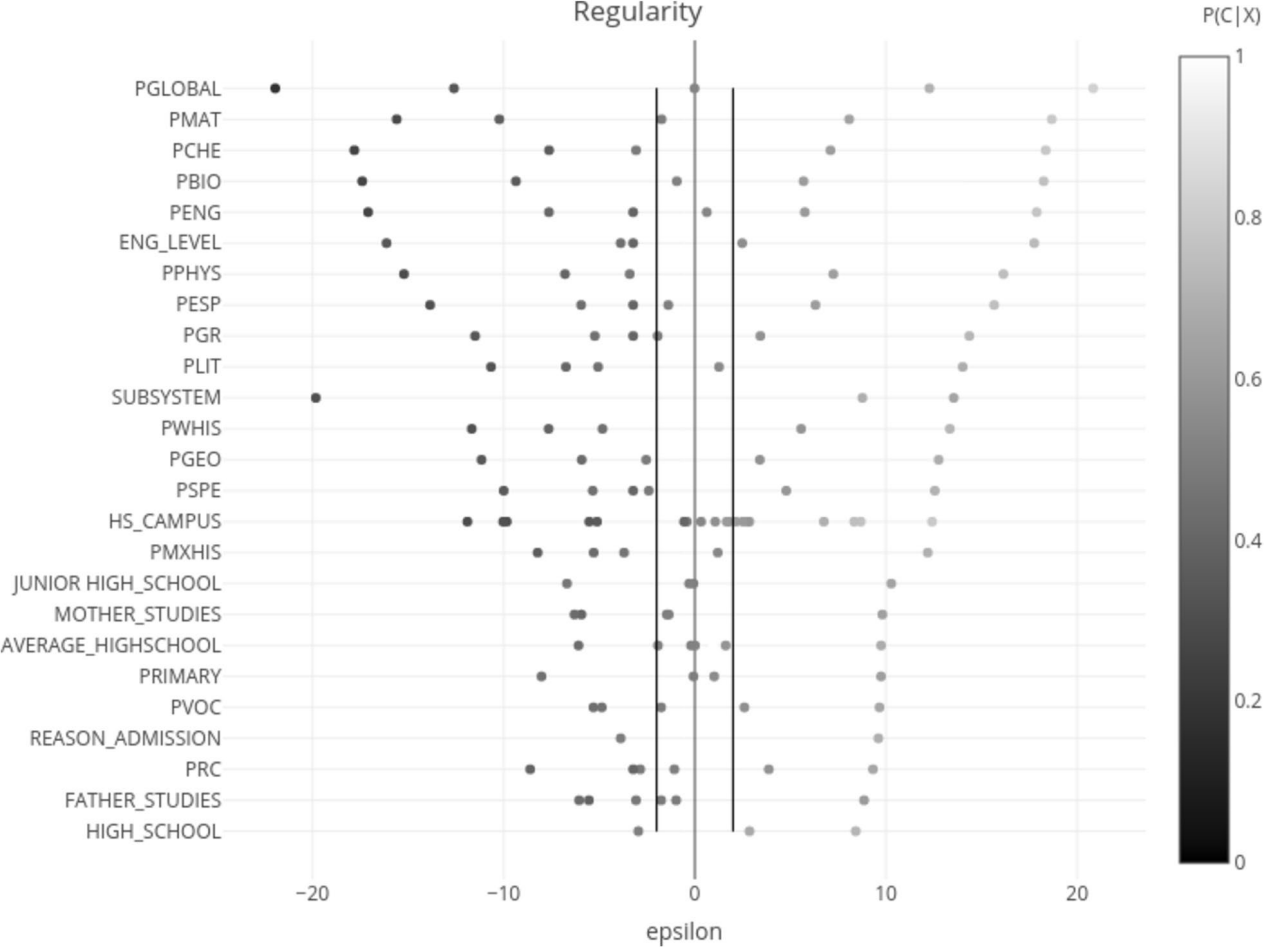
Epsilon results from Naïve Bayes for regularity. *N* = 7,066 from 2011 to 2017 cohorts, UNAM Faculty of Medicine, Mexico. The figure is divided in two parts: on the right are the results for regularity and on the left those for irregularity. The variables are depicted along the vertical axis, ordered according to their relevance based on epsilon: those with the largest epsilon values appear at the top, which reflects greater statistical significance. The two vertical lines represent the points where epsilon is equal to 2 and − 2, respectively. Positive epsilon values indicate the categories which favour belonging to the target group (right side), while the negatives ones the categories which do not (left side). The colour intensity of the dots represents the probability of belonging to the target group, with lighter dots representing those categories with a probability closer to 1, and darker ones with a probability closer to 0

**Fig. 2:**
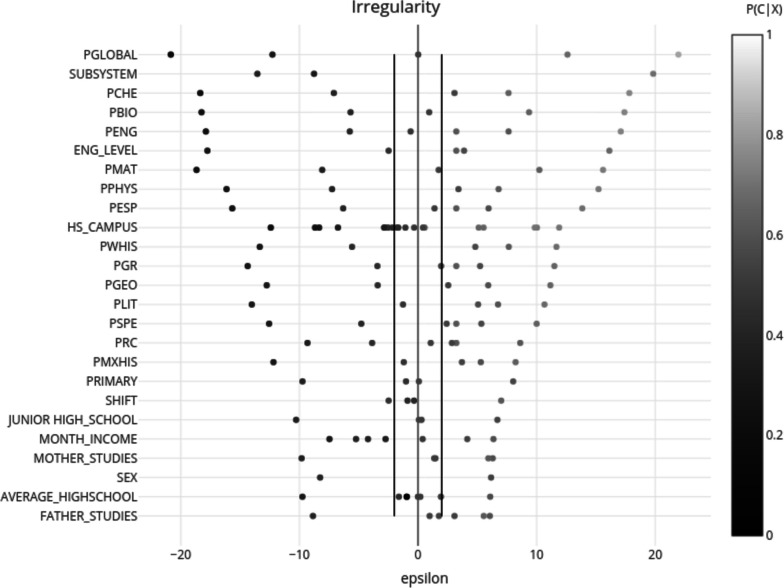
Epsilon results from Naïve Bayes for irregularity. *N* = 7,066 from 2011 to 2017 cohorts, UNAM Faculty of Medicine, Mexico. The figure is divided in two parts: on the right are the results for regularity and on the left those for irregularity. The variables are depicted along the vertical axis, ordered according to their relevance based on epsilon: those with the largest epsilon values appear at the top, which reflects their greater statistical significance. The two vertical lines represent the points where epsilon is equal to 2 and − 2, respectively. Positive epsilon values indicate the categories which favour belonging to the target group (right side), while the negatives ones the categories which do not (left side). The colour intensity of the dots represents the probability of belonging to the target group, with lighter dots representing those categories with a probability closer to 1, and darker ones with a probability closer to 0

A graphic summary of the results from the Naïve Bayes model’s variables analysis for regularity and irregularity is shown in Figs. 1 and 2, respectively.

## Discussion

Both models (ANN and NB) achieved an accuracy around 70% when classifying irregular students, which is consistent with the findings of published studies from different disciplines [21, 24–30, 32, 34, 35, 40, 45–49]. Slight differences (not greater than 0.5) were found in sensitivity, accuracy, and specificity among the models, with ANNs performing better. ANNs achieved a considerably higher positive predictive value than the Naïve Bayes models; in contrast, the latter model reported higher negative predictive values.

The percentage of correct answers in the knowledge diagnostic exam was identified as the most relevant factor for predicting regularity and irregularity, this finding is similar to reports from other institutions, where it was found that one of the most important predictors for academic success was the students’ results in admission exams and their previous academic grades [3, 19]. As Li and colleagues showed in the Australian setting, admission examinations that measure academic achievement can provide important information to be used by medical schools [3]. In our case, we used a standardised diagnostic exam that has a long history in our institution, which can be a source of valuable information to identify in a timely manner students that have academic difficulties, beyond its routine use of providing the results to the institutional authorities.

When the variables were analysed based on their epsilon value, there were differences on how the most relevant variables for prediction were ranked when predicting regularity compared to irregularity. Mainly the variables related to their previous school (type and campus), as well as to the student’s scores in math, literature, grammar, and reading comprehension. The former group of variables has great relevance for predicting students’ irregularity, and the latter, in predicting regularity. Additionally, there were attributes that had a higher predictive value when predicting regularity, such as the reason for admission and the percentage of correct answers in the Spanish vocabulary section; and in the case of predicting irregularity, the attributes were gender, and the student’s family income.

The relevance of a student’s prior knowledge in chemistry, biology, physics, mathematics, and topics related to mastery of Spanish and English is corroborated as a predictor of the student’s success in this medical school. This finding is consistent with previous research [10, 11] and stresses the importance of implementing interventions to strengthen first-year students’ knowledge in these areas. Even though English proficiency was also important, it is necessary to study its apparent relevance to verify whether its predictive relevance is related to the use of English for studying throughout the program, or whether it functions as a proxy of the family’s socio-economic status, since in Mexico this factor may be associated with more opportunities to study a second language.

The study results raise the question of why high school type is a good predictor of irregularity, but less impactful when predicting regularity. A hypothesis could be that the nature of the curriculum creates a disadvantage for some of the students in these high school subsystems. Since students choose which courses to study in their high school last year from a variety of courses, a student in a type B school can graduate without taking biology, chemistry or advanced physics. In contrast, those in type A have to take the classes prescribed for the undergraduate program they wish to study. Additionally, in type B, students only study one course in advanced mathematics during their last year of secondary school and they are not required to study English. Conversely, students in a type A program, have completed two classes in advanced mathematics as well as English.

### Possible uses of the models

When designing an intervention, if the main priority is to identify a large volume of at-risk students, the ANN models would be recommended since they are more precise, which will result in better sensitivity and specificity. If the school administrators want to explore what factors might be influencing students’ performance and identify students that might be at-risk, the Naïve Bayes model would be more useful in this scenario. Lastly, if an intervention will focus on giving personalised support to students, ANNs would be recommended since they had a higher positive predictive value.

It is worth mentioning that interventions aided with machine learning algorithms may have important drawbacks [50]. None of the models achieved a perfect accuracy, there will be false positives and negatives. Interventions that integrate data analysis in their decision process will have to deal with dilemmas such as: is it more important to make sure that irregular students receive the intervention even though some regular students are included? Or is it better to optimise resources by reducing the probability that regular students are considered in the intervention?

Medical educators and university administrators should explore interventions to improve students’ academic progress, using currently available data analysis methodologies from the very beginning of medical school, while closely monitoring their trajectories. Interventions should have a solid pedagogical basis, grounded in the self-regulated learning and motivation literature [50, 51].

### Limitations

The variables used in these models only contain information related to the student’s academic trajectory and socio-economic status. Additional factors, such as the students’ motivation and interests, their social and academic integration at the university, as well as teacher attributes, were not considered in the study. Biases related to socio-economic factors, culture and gender should be assessed in future studies.

910 Students’ records were not included since they had a significant proportion of missing data, it is possible some factors that could influence the model’s prediction were not considered. However, among the population of excluded students, 48% were irregular at the end of the first year, a proportion similar to the overall population analysed.

## Conclusions

This study found that both ANN and Naïve Bayes methods can be useful for predicting students’ academic achievement in an undergraduate program in medicine, based on information about their prior knowledge and socio-demographic factors. Although ANN offered slightly superior results, Naïve Bayes made it possible to obtain an in-depth analysis of how the different variables influenced the model.

By analysing the epsilon values from the Naïve Bayes model, students’ prior academic knowledge was identified as the most important prediction factor in first year medical students. This finding could be useful when designing programs to help students with poor academic performance or that have a high risk of dropping out. Such programs could entail courses delivered to the students after they enrol but before they start their academic program. This type of initiative aligns with a fundamental factor for achieving meaningful learning, to have a vast body of knowledge to be able to connect new information with previous learning.

The use of educational data mining techniques and machine learning classification techniques have great potential in medical education, educators need to familiarize themselves with the basic concepts of this area and begin to explore their use for educational activities, assessment and decision making.

## Data Availability

The datasets generated and analysed during the current study are available in the Educational Innovation Repository of the National Autonomous University of Mexico, [http://www.innovacioneducativa.unam.mx:8080/jspui/handle/123456789/5524]

## Abbreviations

ANN: Artificial Neural networks
EDM: Educational Data Mining
LMS: Learning Management System

## Footnotes

1 In Mexico, if students fail a course final exam, they have the opportunity to take it again (resit or makeup exam). In the database, the former is identified as the regular exam, and the latter, as the resit exam.
